# A Novel Anaesthetical Approach to Patients with Brugada Syndrome in Neurosurgery

**DOI:** 10.1155/2013/280826

**Published:** 2013-05-28

**Authors:** Pietro Paolo Martorano, Edoardo Barboni, Giovanni Buscema, Alessandro Di Rienzo

**Affiliations:** ^1^Head of Neuroanesthesia Unit, Ospedali Riuniti, Via Conca 71 - 60126 Ancona, Italy; ^2^Clinic of Anesthesia and Intensive Care Unit, Department of Emergency, Ospedali Riuniti, Via Conca 71 - 60126 Ancona, Italy; ^3^Anesthesia and Intensive Care Unit, AOU G. Rodolico, Via S. Sofia 78 - 95123 Catania, Italy; ^4^Department of Neurosurgery, Università Politecnica delle Marche, Ospedali Riuniti, Via Conca 71 - 60126 Ancona, Italy

## Abstract

Brugada syndrome (BrS) is one of the most common causes of sudden death in young people. It usually presents with life-threatening arrhythmias in subjects without remarkable medical history. The need for surgical treatment may unmask BrS in otherwise asymptomatic patients. The best anaesthesiological treatment in such cases is matter of debate. We report a case of neurosurgical treatment of cerebello pontine angle (CPA) tumor in a BrS patient, performed under total intravenous anesthesia (TIVA) with target controlled infusion (TCI) modalities, using midazolam plus remifentanil and rocuronium, without recordings of intraoperative ECG alterations in the intraoperative period and postoperative complications.

## 1. Introduction

BrS is a rare dominant autosomal disease with incomplete penetrance, first described in 1992 by P. Brugada and J. Brugada [1]. More common in men than in women, it is typically diagnosed during the fourth decade of life, and it is caused by a genetic mutation affecting the ion channels of the cardiac conduction system. The typical clinical correlate is a coved ST segment elevation in the right precordial leads that can occur with or without an incomplete right bundle branch block. 

Owing to its phenotypic variability, clinical manifestations of BrS are protean, including syncope or spontaneous ventricular arrhythmias that can lead to a sudden death [2, 3], which all may be elicited in such peculiar situations (vagal tone increase, fever, and electrolytes disorder) or by peculiar drugs administration including some anaesthetics [4]. There is still no consense on which the golden standard should be in case of general anaesthesia in these cases, especially because of the low prevalence of BrS, the absence of large prospective study, and the different anaesthesiological needs according to different surgical specialties. Existing guidelines derives from theoretical model based on the pathophysiological mechanism of BrS and from case series regarding a small number of patients. As regards the use of intravenous anesthetics in patients with BrS, propofol, and midazolam wase successfully used in different procedures [5, 6]. Propofol is a short acting, intravenous hypnotic, that ensures fast onset and rapid recovery of anesthesia, reducing PONV (postoperative nausea and vomiting). It represents the hypnotic of choice for TIVA/TCI use in neurosurgery, due to his low impact on CBF and the ability to mantain cerebral autoregulation, however, allowing a rapid recovery of the cognitive function at the end of the procedure. The recommendation to avoid it in patient with BrS is based on it is potential to expose patients to the risk of developing malignant arrhythmias like ventricular tachycardia (VT) or fibrillation followed by death [7, 8] and the acquired Brugada-like electrocardiographic (ECG) changes following high-dose propofol infusion over prolonged periods of time [9].

Midazolam is a short-acting benzodiazepine (BZD) [10] commonly used for sedation or induction in anesthesia, that appears to be safe in patient with BrS. It has a low metabolic, hemodynamic impact, and recent evidences of neuroprotective effects [11]. Its pharmacokinetics change significantly during continuous infusion [12], resulting in prolonged duration of action and delayed recovery. In this setting it could be worthwhile to use a target controlled infusion (TCI) system, like navigator suite GE, to provide a better dosage adjustments on an real time basis to maintain adequate hypnosis and rapid recovery especially during neurosurgery procedure.

We report our experience with the intraoperative use of midazolam in a BrS patient undergoing a neurosurgery procedure for the removal of a large tumor of the cerebellopontine angle (CPA). 

## 2. Case Presentation

A 44-year-old male patient, BMI 23.45, ASA score 2, was scheduled for the removal of a cerebellopontine angle tumor. At neurological examination he presented with incomplete left VIIth cranial nerve deficit (House Brackmann grade II), dysphagia, lateral left gaze diplopia. MRI examination evidenced a 4 cm diameter tumor of the left cerebellopontine angle, with brainstem compression/dislocation, suspect for a meningioma. The patient reported a recent history of severe cardiac symptoms (arrhythmias and cardiac arrest) with full recovery and a diagnosis of Brugada syndrome based on the ECG pattern (type 1).

Previously had undergone appendectomy, without report of cardiological complication.

Preoperative blood analysis and serum electrolytes were normal. 

## 3. Intraoperative Anaesthesiological Management

Peripheral venous access was obtained by a 18 Ga needle, then a 0.9% NaCl infusion was started. Before induction, adhesive plaques connected to a biphasic defibrillator with pacemaker (PM) features were applied to the patient. During surgery, electrocardiography, invasive blood pressure, pulse oximetry, body temperature, and neuromuscular blockade degree were continuously recorded, and the depth of anaesthesia was monitored by entropy (GE Healthcare, Finland) [13].

Total intravenous anaesthesia was administered using a Fresenius Orchestra Base Primea (Fresenius Kabi, France) linked to a multimodal navigator suite GE (GE Healthcare Finland).

**Figure 1:**
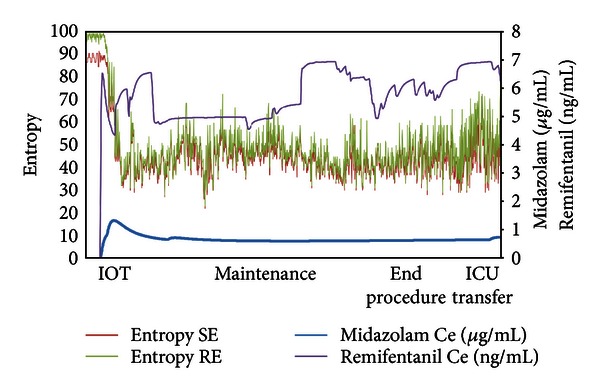
This figure represents the trend of the concentrations of hypnotic (midazolam) and opioid (remifentanil) and their correlation with the adequacy of the anesthesia plan obtained by entropy (state entropy (SE), response entropy (RE)).

General anesthesia was induced by intravenous midazolam (0.2 mg/kg) plus remifentanil (1 *μ*g/kg) followed by rocuronium (0.9 mg/kg), before intubation. Mechanical ventilation (tidal volume 6 mL/kg; FiO_2_ O_2_ 50%) was started and settled to an EtCO_2_ of 30–35 mmHg. The anaesthesiological plan was maintained by infusion of midazolam (Effect Site concentration: Ce 0.68 ± 2.25 ng/mL) and remifentanil (effect site concentration: Ce 5.60 ± 1.23 ng/mL) in order to maintain state entropy <55 (SE) (Table 1) (Figure 1).

Data were collected every 10 seconds by a dedicated software (S5 collect GE Healthcare, Finland) and stored in Excel files.

Crystalloids were infused at a speed of 6 mL/kg/h. Surgery lasted approximately 240 min. No shiver episodes, psychomotor agitation, respiratory crises, or any cardiovascular issue or were observed either at induction and during maintenance. At the end of surgery the patient was transferred to the ICU, and the following postoperative course was uneventful.

## 4. Discussion

BrS patients are at elevated risk of death, this condition being considered the most prevalent cause of sudden cardiac death in a young population [3]. Such disorder raises specific concerns as anesthesiologists routinely administer drugs that interact with cardiac ion channels, potentially triggering the development of malignant arrhythmias. Previous surgeries performed under uneventful general anaesthesia do not lower risks of subsequent adverse events, so that an accurate preoperative evaluation of symptoms and a multidisciplinary approach are required for a well-planned management. There is a lack of evidence in the literature about the most suitable drugs and their side effects during anaesthesiological treatment of BrS. 

Absence of prospective studies combined with low prevalence of BrS hinder exact guidelines creation and recommendations are derived exclusively from theoretical models based on disease pathophysiology and direct observations from case reports and short series [7].

Propofol, a phenol derived drug, represents the choice hypnotic for TIVA/TCI use in neurosurgery, due to his low impact on CBF, fast offset, and low PONV. Unfortunately, it is burdened from the potential to alter ion channel function inducing Brugada-like ECG abnormalities, which implies an increased risk of malignant arrhythmias in BrS patients [4, 5]. The literature supports the recommendation to avoid propofol administration either during induction or continuous infusions in BrS patients. 

Midazolam is a largely diffused benzodiazepine with no adverse cardiac effects. Nonetheless, its use is penalized by a trend toward accumulation and by the following unpredictable recovery. In the case reported, such difficulty was to be overcome by using a TIVA/TCI midazolam/remifentanil pump (pharmacokinetic/pharmacodynamic (PK/PD) model) coupled with monitoring of hypnosis depth by spectral entropy. This allowed a closer titration of drug administration based on calculation of the predicted synergistic effects of the two drugs and on the feedback of brain electrical activity. Factors that might exacerbate ST segment elevations and lead to dysrhythmias, as hyperthermia, bradycardia, electrolyte imbalances, hyper/hypokalemia, and hypercalcemia, could be rapidly identified and corrected, as well as autonomic imbalance and fast postural changes.

Current recommendations about patients diagnosed with a hereditary arrhythmogenic syndrome, mostly derive from expert's opinions [5], a careful evaluation before operation, and close intraoperative hemodynamic monitoring are advised. The specific anaesthesiological implications and prompt therapeutic interventions required in these cases make the perioperative management of these patients a challenge for the anesthesiologist. 

Adequate anaesthesia and analgesia, propofol avoidance, maintenance of homeostasis and continuous monitoring in the postoperative period for at least 24 h are strongly recommended.

More studies about anaesthetic drugs and molecular biology are necessary to choose the optimal drugs and anaesthesiological management in these patients. A multidisciplinary approach is recommended for a well-planned perioperative management tailored to individual patient's needs. In this context we believe that the use of anaesthesia multimodality systems ensures better intraoperative conditions while offering assurances of safety and efficacy.

## Figures and Tables

**Table 1:** The average values of the concentrations of the drugs infused in target controlled infusion (TCI), the average values of Entropy and heart rate and mean arterial pressure.

	Mean ± St. dev.
Remifentanil Ce induction (ng/mL)	5.78 ± 0.00
Remifentanil Ce intraoperative (ng/mL)	5.60 ± 1.23
Midazolam Ce induction (*µ*g/mL)	1.23 ± 0.00
Midazolam Ce intraoperative (*μ*g/mL)	0.68 ± 2.25
State entropy (SE) intraoperative	45.51 ± 9.98
Response entropy (RE) intraoperative	49.29 ± 12.16
Heart rate (bpm)	79.13 ± 5.41
Mean arterial pressure (mmHg)	67.34 ± 14.90

Values are presented as mean ± standard deviation.
